# Time-lagged effects of social support on exercise adherence among Korean college students: serial mediation of self-efficacy, athletic identity, and intrinsic motivation

**DOI:** 10.3389/fpsyg.2026.1795823

**Published:** 2026-04-16

**Authors:** Yang Zhao, Xiangnan Li, Yubo Zhou, Du Wang

**Affiliations:** 1 Seoul School of Integrated Sciences and Technologies (aSSIST), Seoul, Republic of Korea; 2 School of Kinesiology and Physical Education, Zhengzhou University, Zhengzhou, China; 3 ALFA University College (AUC), Subang Jaya, Selangor, Malaysia

**Keywords:** athletic identity, college students, exercise adherence, intrinsic motivation, longitudinal study, self-efficacy, social support

## Abstract

**Introduction:**

Physical activity levels decline substantially during the college years, yet the psychological mechanisms linking social support to sustained exercise adherence remain insufficiently understood through longitudinal research. Grounded in Social Cognitive Theory, this study examined the time-lagged mediating mechanisms through which social support influences exercise adherence among Korean college students.

**Methods:**

A three-wave longitudinal design was employed with 950 Korean college students (47.1% male; *M*_*age*_ = 20.3 years). Social support and baseline exercise adherence were assessed at T1 (baseline); exercise self-efficacy and athletic identity at T2 (2 weeks later); and intrinsic motivation and exercise adherence at T3 (4 to 6 weeks later). Time-lagged mediation was tested using structural equation modeling with bias-corrected bootstrap confidence intervals (5,000 resamples).

**Results:**

After controlling for baseline exercise adherence, the total effect of social support on T3 exercise adherence was small (β = 0.06, *p* = 0.061), while the direct effect was negligible (β = 0.00, *p* = 0.924). The total indirect effect was significant (β = 0.05, 95% CI [0.030, 0.073]), consistent with an indirect-only mediation pattern. Three significant indirect pathways were identified: (1) social support → self-efficacy → exercise adherence (effect = 0.029, 95% CI [0.013, 0.049]); (2) social support → self-efficacy → intrinsic motivation → exercise adherence (effect = 0.013, 95% CI [0.007, 0.021]); and (3) social support → athletic identity → intrinsic motivation → exercise adherence (effect = 0.004, 95% CI [0.002, 0.008]).

**Discussion:**

These findings provide time-lagged evidence that social support is associated with exercise adherence primarily through enhancing self-efficacy, which influences adherence both directly and through intrinsic motivation. Athletic identity contributed indirectly via intrinsic motivation rather than directly predicting adherence. The results support Social Cognitive Theory's emphasis on self-efficacy as a key mediator linking environmental factors to health behaviors, and suggest that interventions combining social support enhancement with self-efficacy building may be most effective for promoting sustained physical activity among college students.

## 1 Introduction

The transition to college represents a critical developmental period during which physical activity habits are either consolidated into lifelong patterns or gradually abandoned (Liu et al., 2023). This period is particularly consequential because behavioral patterns established during early adulthood tend to persist throughout the lifespan, with profound implications for long-term health trajectories. Despite well-documented benefits of regular physical exercise—including enhanced cardiovascular health, improved psychological wellbeing, better academic performance, and reduced risk of chronic diseases (Brown et al., 2024; Liu Y. et al., 2024; Mahindru et al., 2023)—epidemiological evidence reveals a troubling paradox: physical activity levels decline precipitously during the very years when young adults have the greatest opportunity to establish healthy habits (Li et al., 2024a; Hu et al., 2022).

This decline is particularly pronounced among East Asian college students. The World Health Organization recommends that adults aged 18–64 engage in at least 150–300 min of moderate-intensity aerobic physical activity per week to reduce the risk of cardiovascular disease, type 2 diabetes, and depression (World Health Organization, 2020). However, in China and Korea, recent surveys indicate that fewer than 30% of college students meet these recommended physical activity guidelines (Hu et al., 2022; Kim et al., 2023). In South Korea specifically, national data suggest that only 24.7% of young adults aged 19–29 meet the aerobic physical activity guidelines, and this proportion has continued to deteriorate following the COVID-19 pandemic (Korea Disease Control and Prevention Agency, 2023). The consequences of physical inactivity extend beyond individual health: insufficient physical activity among college students is associated with increased rates of obesity, metabolic syndrome, anxiety, and depression (Mahindru et al., 2023; World Health Organization, 2020), and contributes to rising healthcare costs, reduced workforce productivity, and diminished quality of life across populations (Brown et al., 2024). Understanding why some students maintain regular exercise while others abandon physical activity—and identifying the modifiable mechanisms that promote sustained engagement—has therefore become a recognized public health priority (Li et al., 2024a; Brown et al., 2024).

Central to this inquiry is the concept of exercise adherence. Unlike isolated measures of physical activity captured at a single time point, exercise adherence reflects the sustained, regular participation in physical activities over time, encompassing both behavioral consistency and psychological commitment (Wang et al., 2016). Exercise adherence captures individuals' emotional investment in physical activity, the volitional effort they dedicate to maintaining routines, and their persistence in the face of obstacles (Kovács and Szakál, 2024). Notably, emerging research emphasizes that even short bouts of physical activity accumulated throughout the day can produce meaningful health benefits, and current guidelines increasingly recommend that individuals integrate movement into their daily routines in addition to meeting minimum aerobic activity thresholds (World Health Organization, 2020; Stamatakis et al., 2022). Given that the health benefits of physical activity are dose-dependent and cumulative, exercise adherence—the capacity to maintain regular physical activity over time—rather than occasional participation, constitutes the critical behavioral outcome for health promotion (André et al., 2024).

Identifying the factors that determine whether college students develop and maintain exercise adherence is therefore essential. Theoretical models of health behavior change consistently identify two categories of determinants: environmental factors that provide resources and constraints, and individual psychological factors that regulate motivation and self-regulation (Bandura, 2004; Brown et al., 2024). Among environmental factors, social support has emerged as one of the most robust predictors of physical activity behavior. Social support encompasses the perception and actuality of being cared for, having assistance available from others, and belonging to a supportive social network (Ye, 2008). For college students navigating the competing demands of academic work, social relationships, and career preparation, social support from family members, friends, classmates, and exercise companions may provide the encouragement and resources necessary to prioritize physical activity (Liu W. et al., 2024; Mema et al., 2022; Tian and Shi, 2022).

However, while the association between social support and exercise adherence is well-established (Mema et al., 2022; Tian and Shi, 2022; Brown et al., 2024), the mechanisms through which social support translates into sustained behavior remain incompletely understood. Social Cognitive Theory (SCT) posits that environmental factors do not directly determine behavior but rather operate through their effects on cognitive mediators (Bandura, 1986, 2004). This theoretical perspective highlights the need to identify the specific psychological pathways through which social support influences exercise adherence, to determine whether multiple mechanisms operate in parallel or function sequentially, and to establish the temporal ordering necessary to support inferences about mediation processes through longitudinal research.

The present study addresses these gaps by employing a three-wave longitudinal design to examine the time-lagged mediating mechanisms linking social support to exercise adherence among Korean college students. Specifically, we test whether exercise self-efficacy—confidence in one's ability to maintain exercise routines—and athletic identity—the degree to which individuals incorporate the exerciser role into their self-concept (Dong, 2017)—serve as parallel mediators, and whether intrinsic motivation functions as a downstream mediator in serial pathways. By measuring the predictor, mediators, and outcome at separate time points and controlling for baseline exercise adherence, this design enables stronger inferences about temporal precedence than the cross-sectional approaches that have dominated previous research.

## 2 Theory and hypotheses

### 2.1 Theoretical framework: social cognitive theory

Social Cognitive Theory (SCT), developed by (Bandura 1986), provides the primary theoretical framework for understanding how environmental factors influence health behaviors through psychological mechanisms. SCT conceptualizes human functioning as emerging from the dynamic, reciprocal interaction among three classes of determinants: personal factors (cognitive, affective, and biological processes), behavioral factors (actions and their consequences), and environmental factors (social and physical contexts) (Bandura, 1986, 2004). This triadic reciprocal causation model emphasizes that individuals are neither passive recipients of environmental influences nor autonomous agents acting independently of context, but rather active participants in a continuous process of mutual influence among person, behavior, and environment (Bandura, 2004; Schunk and DiBenedetto, 2020).

A central tenet of SCT—and one particularly relevant to understanding exercise adherence—is that environmental influences shape behavior primarily through their effects on self-regulatory mechanisms (Bandura, 1986, 2004). Among these mechanisms, self-efficacy occupies a pivotal position as the foundation of human agency (Bandura, 2004). Self-efficacy refers to individuals' beliefs about their capabilities to execute courses of action required to produce desired outcomes (Bandura, 1986). These beliefs are not merely passive reflections of past performance but rather active determinants of future behavior: individuals with high self-efficacy set more challenging goals, exert greater effort, persist longer when facing obstacles, and recover more quickly from setbacks (Schunk and DiBenedetto, 2020).

In the exercise domain, self-efficacy reflects confidence in one's ability to overcome barriers to physical activity, maintain consistency in exercise routines, and persist when facing challenges such as fatigue, time constraints, or competing demands (Lee et al., 2009). According to SCT, self-efficacy beliefs are constructed from four principal sources of information: mastery experiences (successful performance accomplishments that provide direct evidence of capability), vicarious experiences (observing similar others succeed), verbal persuasion (encouragement and feedback from significant others), and physiological and affective states (interpreting bodily sensations as indicators of capability) (Bandura, 1986). This framework suggests multiple pathways through which social support may enhance exercise self-efficacy. Supportive others provide verbal encouragement that conveys confidence in the individual's capabilities, serving as a form of verbal persuasion. They also model successful exercise behaviors that enable vicarious learning. Additionally, social support offers practical assistance that facilitates mastery experiences, and creates positive emotional contexts that promote favorable interpretations of physiological states (Caetano et al., 2020; Ke et al., 2025).

Beyond self-efficacy, SCT recognizes that behavior is influenced by additional cognitive and motivational factors (Bandura, 2004; Schunk and DiBenedetto, 2020). Among the cognitive factors, outcome expectations—beliefs about the likely consequences of actions—shape behavioral choices, and self-regulatory capabilities—including goal-setting, self-monitoring, and self-evaluation—determine whether intentions translate into sustained action. Among the motivational factors, intrinsic motivation arising from inherent interest and enjoyment influences the quality and persistence of behavioral engagement (Bandura, 2004). These cognitive and motivational processes do not operate in isolation but rather form interconnected pathways through which environmental factors ultimately influence behavior (Bandura, 1986; Schunk and DiBenedetto, 2020).

Applying this framework to exercise adherence suggests a theoretical model in which social support (an environmental factor) influences exercise adherence (a behavioral outcome) through its effects on intermediate psychological mechanisms. Specifically, social support may enhance self-efficacy beliefs, which in turn promote both direct effects on exercise adherence and indirect effects through motivational processes. This reasoning leads to testable hypotheses about mediation pathways that can be examined using longitudinal designs capable of establishing temporal precedence.

### 2.2 Social support and exercise adherence: the direct pathway

Social support represents a key environmental factor in SCT's triadic model, providing resources that facilitate health behavior change. Empirical research has consistently demonstrated positive associations between social support and exercise-related outcomes among college students (Mema et al., 2022; Tian and Shi, 2022). A recent systematic review examining influences on university students' physical activity identified social support as one of the most frequently reported facilitators across diverse samples and methodological approaches (Brown et al., 2024). Prospective studies have further shown that social support predicts changes in physical activity over time, supporting its role as an antecedent rather than merely a correlate of exercise behavior (Liu W. et al., 2024).

The positive relationship between social support and exercise adherence can be understood through multiple complementary mechanisms. From a resource perspective, social support provides tangible assistance that reduces barriers to exercise: friends may offer transportation to fitness facilities, family members may accommodate exercise schedules, and exercise companions may provide equipment or expertise (Tian and Shi, 2022). From an emotional perspective, supportive relationships buffer stress and enhance psychological wellbeing, creating conditions favorable for engaging in health-promoting behaviors rather than coping behaviors (Liu W. et al., 2024). From a normative perspective, social networks define appropriate behaviors and create contexts in which exercise is valued, expected, and reinforced (Wang et al., 2016). Importantly, SCT recognizes that social influences operate even for individuals who prefer to exercise alone. The social environment may shape exercise behavior indirectly by influencing self-efficacy beliefs, outcome expectations, and normative perceptions that sustain independent exercise routines (Bandura, 2004). Thus, social support need not involve direct companionship during exercise; rather, it may function through cognitive and motivational channels that support autonomous engagement in physical activity.

Beyond cross-sectional evidence, several longitudinal and experimental studies have examined the social support–exercise adherence association. Randomized controlled trials (RCTs) have demonstrated that social support interventions can increase physical activity levels. For example, (Dishman et al. 2005) found in a school-based RCT that interventions incorporating social support components significantly improved physical activity among adolescent girls, and (Lubans et al. 2008) reported in a systematic review that social support interventions produced small-to-moderate effects on youth physical activity. Among longitudinal studies, (Tian and Shi 2022) found that social support predicted exercise adherence among Chinese college students during the COVID-19 pandemic, providing evidence for temporal precedence. However, their two-wave design limited examination of sequential mediating mechanisms and did not control for baseline exercise adherence—a critical omission given the strong autoregressive relationship between earlier and later behavior. The present study extends this body of work by employing a three-wave design that enables examination of time-lagged effects while controlling for baseline exercise adherence, providing a more rigorous test of whether social support predicts later adherence above and beyond baseline levels. Accordingly, we hypothesize:

**Hypothesis 1:** Social support at T1 is expected to show a small positive association with exercise adherence at T3, after controlling for baseline exercise adherence.

### 2.3 The mediating role of exercise self-efficacy

If SCT is correct that environmental factors influence behavior through cognitive mediators, then the association between social support and exercise adherence should be at least partially explained by intermediate psychological variables—most prominently, self-efficacy. Self-efficacy has been identified as one of the strongest and most consistent psychological predictors of exercise behavior in the empirical literature (Caetano et al., 2020; Brown et al., 2024). Meta-analytic evidence indicates that self-efficacy accounts for substantial variance in physical activity across diverse populations, age groups, and cultural contexts (Lee et al., 2009). Longitudinal studies have further demonstrated that self-efficacy predicts later physical activity above and beyond baseline levels, supporting its role as a prospective predictor rather than merely a consequence of exercise behavior (Li et al., 2024a).

The theoretical rationale for social support enhancing self-efficacy derives directly from Bandura's (1986) specification of self-efficacy sources. Social support operates through at least three of these sources (Bandura, 1986; Schunk and DiBenedetto, 2020). First, supportive others provide verbal encouragement and positive feedback that convey confidence in the individual's exercise capabilities—a form of verbal persuasion that can strengthen efficacy beliefs, particularly when the source is credible and trustworthy (Bandura, 2004). Second, supportive relationships create opportunities for observational learning: exercising with friends or family members who model successful physical activity provides vicarious experiences that demonstrate the attainability of regular exercise (Schunk and DiBenedetto, 2020). Third, social support facilitates mastery experiences by providing instrumental assistance (e.g., transportation, childcare, flexible scheduling) that removes barriers and enables successful exercise participation (Schunk and DiBenedetto, 2020; Bandura, 1986).

Empirical evidence supports these theoretical pathways through a range of study designs. Meta-analyses have confirmed that self-efficacy is one of the strongest correlates of physical activity across populations, with moderate-to-large effect sizes (Ashford et al., 2010; Williams et al., 2005). Randomized controlled trials have demonstrated that interventions targeting self-efficacy sources—such as graded mastery experiences, peer modeling, and verbal persuasion—produce significant increases in physical activity behavior (Ashford et al., 2010; McAuley et al., 2011). In the exercise domain specifically, (Ke et al. 2025) found that physical self-efficacy mediated the relationship between perceived transformational leadership of physical education teachers and exercise adherence among college students. (Sánchez-García et al. 2024) demonstrated that teachers' transformational leadership influenced students' motor self-efficacy, which in turn predicted intentions for physical activity. These findings from both experimental and observational studies converge to suggest that social-environmental factors influence exercise behavior through their effects on efficacy beliefs, consistent with SCT predictions.

The link between self-efficacy and exercise adherence is similarly well-documented. Individuals with higher exercise self-efficacy report greater intention to exercise, more frequent physical activity participation, and better maintenance of exercise routines over time (Caetano et al., 2020). Self-efficacy influences adherence through multiple mechanisms: individuals with high self-efficacy set more challenging exercise goals, maintain effort when facing barriers such as time constraints or fatigue, develop more effective self-regulatory strategies, and recover more quickly from lapses in their exercise routines (Lee et al., 2009).

Integrating these relationships, the mediation pathway from social support through self-efficacy to exercise adherence is well-grounded in SCT and has received empirical support from multiple lines of evidence. Cross-sectional studies have documented this pathway (Ke et al., 2025; Sánchez-García et al., 2024), and intervention studies targeting self-efficacy have demonstrated downstream effects on physical activity behavior (McAuley et al., 2011; Ashford et al., 2010). However, longitudinal evidence establishing the temporal ordering of social support, self-efficacy, and exercise adherence within a single mediational framework remains limited. The present study addresses this gap by measuring social support, self-efficacy, and exercise adherence at separate time points to test temporal precedence. We hypothesize:

**Hypothesis 2:** Exercise self-efficacy at T2 mediates the relationship between social support at T1 and exercise adherence at T3.

### 2.4 The mediating role of athletic identity

While self-efficacy represents a cognitive mechanism focused on perceived capability, identity represents a complementary mechanism focused on self-definition. Athletic identity refers to the degree to which individuals identify with the athlete or exerciser role, incorporating this role into their self-concept (Dong, 2017). Unlike self-efficacy, which pertains to perceived capability for successful exercise, athletic identity pertains to self-definition—whether an individual views being an exerciser as a core aspect of who they are. Identity-based approaches to behavior change suggest that individuals are strongly motivated to act consistently with their self-definitions, making identity a potentially powerful predictor of sustained behavioral engagement (Griban et al., 2020).

The theoretical rationale for including athletic identity alongside self-efficacy derives from the recognition that sustained behavior change involves not only developing confidence and skills but also integrating new behaviors into one's sense of self (Strachan et al., 2010; Griban et al., 2020). When physical activity becomes part of how individuals define themselves—rather than merely something they do instrumentally—exercise takes on intrinsic meaning and value that sustains engagement even when external rewards or supports diminish (Dong, 2017; Strachan et al., 2010). Individuals with strong athletic identities experience inconsistency between their self-concept and inactivity as psychologically aversive, motivating continued engagement even when facing barriers. This mechanism may be particularly relevant for individuals who prefer to exercise alone: even without direct social companionship, a strong exerciser identity can sustain autonomous motivation and behavioral persistence (Strachan et al., 2010).

Social support may strengthen athletic identity through several mechanisms. Supportive others validate the importance of physical activity, communicating that exercise is a valued and meaningful pursuit. Friends, family members, and exercise companions affirm the individual's identity as an exerciser through recognition, praise, and shared participation. Social contexts that value physical activity create normative environments where the exerciser identity is socially recognized and rewarded (Wang et al., 2016). Over time, these social influences may become internalized as part of the individual's core self-definition through processes of reflected appraisal and social reinforcement (Strachan et al., 2010; Griban et al., 2020).

Empirical research supports the link between athletic identity and exercise persistence. Studies have documented positive associations between athletic identity and sustained physical activity engagement (Castillo et al., 2020). However, most research on athletic identity has focused on competitive athletes rather than recreational exercisers, and few studies have examined athletic identity as a mediator linking social-environmental factors to exercise adherence among general populations.

It should be noted that the strength of identity-behavior relationships may vary across populations and contexts (Strachan et al., 2010; Castillo et al., 2020). Athletic identity is likely to be more central and stable among competitive athletes who have invested years in sport participation than among general college students for whom physical activity may be peripheral to their core self-definitions. Additionally, the relatively brief time frame of typical longitudinal studies (weeks to months) may be insufficient to capture identity formation processes that unfold over longer developmental periods. Furthermore, in East Asian cultural contexts where academic achievement is heavily emphasized, athletic self-definitions may compete with academic identities for psychological centrality, potentially attenuating identity-based effects on exercise behavior. These considerations suggest that while athletic identity may theoretically serve as a mediator, its effects in general college student populations may be modest compared to those observed among dedicated athletes.

The present study addresses this gap by examining athletic identity as a parallel mediator alongside self-efficacy, testing whether social support influences exercise adherence through identity-based mechanisms that complement efficacy-based mechanisms. Given the boundary conditions noted above, we test this pathway as a theoretically plausible but potentially modest mechanism:

**Hypothesis 3:** Athletic identity at T2 may mediate the relationship between social support at T1 and exercise adherence at T3, though effects are expected to be weaker than self-efficacy pathways.

### 2.5 Serial mediation through intrinsic motivation

The hypotheses presented thus far propose that self-efficacy and athletic identity function as parallel mediators linking social support to exercise adherence. However, theoretical considerations suggest that these proximal psychological resources may also influence behavior through their effects on downstream motivational processes—specifically, intrinsic motivation.

Intrinsic motivation refers to engaging in activities for their inherent satisfaction, enjoyment, and interest rather than for external rewards or pressures (Li et al., 2024b). Self-determination theory distinguishes intrinsic motivation from extrinsic forms of motivation and identifies intrinsic motivation as the most autonomous and sustained form of behavioral regulation (Castillo et al., 2020). In the exercise domain, intrinsic motivation has been consistently associated with greater physical activity participation and better long-term adherence: individuals who exercise because they genuinely enjoy it persist longer than those who exercise primarily for external reasons such as weight loss or social approval (Li et al., 2024b; Chen et al., 2013).

SCT and self-determination theory converge in proposing that self-efficacy and identity serve as foundations for intrinsic motivation (Bandura, 2004; Castillo et al., 2020). According to (Bandura 2004), self-efficacy beliefs influence motivational processes by shaping outcome expectations, goal setting, and causal attributions. Individuals with high self-efficacy anticipate success and enjoyment from exercise, set challenging but attainable goals that promote engagement, and attribute outcomes to controllable factors—conditions that foster intrinsic motivation and sustained interest (Schunk and DiBenedetto, 2020). Similarly, when physical activity is integrated into one's identity, engagement becomes self-congruent and personally meaningful. Activities that express and affirm one's identity are experienced as inherently satisfying, promoting intrinsic motivation (Khan et al., 2022).

Research has examined these sequential relationships using various designs. Earlier experimental studies demonstrated that interventions enhancing self-efficacy produced downstream effects on intrinsic motivation and subsequent physical activity participation (McAuley et al., 2011; Williams et al., 2005). More recently, (Ke et al. 2025) found evidence for a serial mediation pathway from transformational leadership to exercise adherence through physical self-efficacy and exercise motivation in their study of college students. This finding suggests that efficacy beliefs may serve as a proximal foundation upon which motivational processes develop, which then directly influence behavior. While the convergence of experimental and correlational evidence supports these sequential pathways, few studies have tested the full serial mediation chain from social support through self-efficacy and motivation to exercise adherence within a single longitudinal framework.

Building on this body of experimental and correlational evidence, we propose two serial mediation pathways linking social support to exercise adherence through intrinsic motivation. In the first pathway, social support enhances self-efficacy, which fosters intrinsic motivation, which in turn promotes adherence. Individuals who receive support develop greater confidence in their exercise capabilities, which leads them to anticipate success and enjoyment, which sustains engagement over time. In the second pathway, social support strengthens athletic identity, which stimulates intrinsic motivation, which promotes adherence. Individuals whose social contexts affirm their identity as exercisers come to view physical activity as self-expressive and personally meaningful, which sustains engagement through inherent interest rather than external pressure.

These serial mediation pathways are consistent with SCT's proposition that environmental factors influence behavior through sequential cognitive and motivational processes. They also suggest a temporal ordering—from environmental support to proximal psychological resources to distal motivational processes to behavior—that can be tested using a three-wave longitudinal design. Given the boundary conditions noted above regarding athletic identity in general student populations, we anticipate that the self-efficacy pathway (H4a) may yield stronger effects than the athletic identity pathway (H4b). We hypothesize:

**Hypothesis 4:** Social support influences exercise adherence through serial mediation pathways: (a) social support enhances self-efficacy, which fosters intrinsic motivation, which in turn promotes exercise adherence; and (b) social support strengthens athletic identity, which stimulates intrinsic motivation, which in turn promotes exercise adherence.

### 2.6 The present study

**Figure 1:**
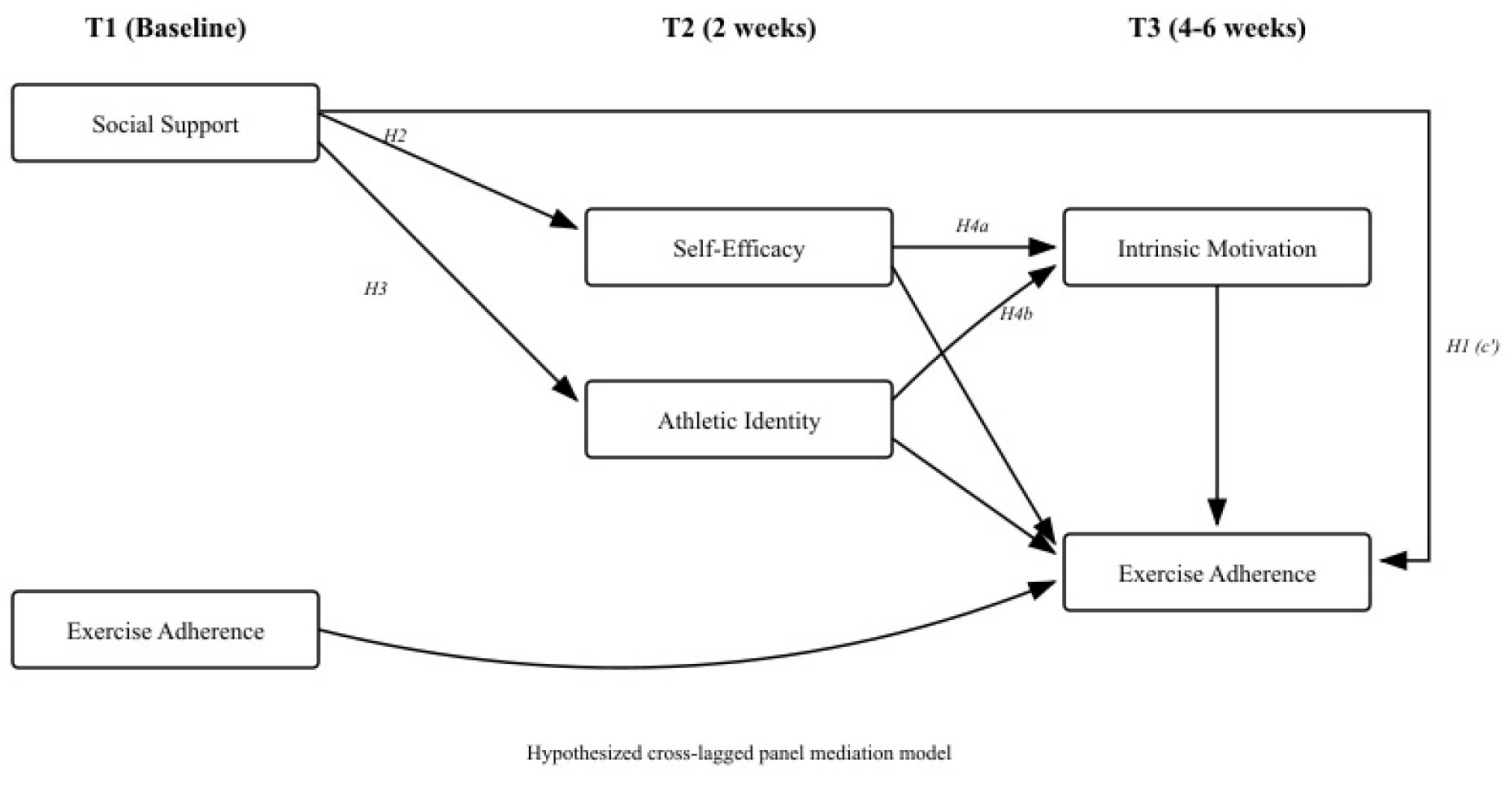
Hypothesized time-lagged mediation model. T1, baseline; T2, 2 weeks after baseline; T3, 4 to 6 weeks after baseline. All paths represent hypothesized relationships. The path labeled *c*′ represents the direct effect of social support on exercise adherence.

The present study extends the existing literature on social support and exercise adherence—which includes cross-sectional, longitudinal, and experimental studies—by integrating multiple mediators within a single time-lagged framework. Although prior research has established individual links in the proposed mediation chain (McAuley et al., 2011; Ke et al., 2025; Tian and Shi, 2022), no study has simultaneously tested the full serial mediation model from social support through self-efficacy and athletic identity to intrinsic motivation and exercise adherence using temporally separated measurements. Our three-wave longitudinal design measures the predictor (social support) at T1, mediators (self-efficacy, athletic identity) at T2, and downstream mediator and outcome (intrinsic motivation, exercise adherence) at T3. Although intrinsic motivation and exercise adherence are assessed concurrently at T3, this design enables stronger inferences about temporal precedence for the initial stages of the hypothesized mediation sequence than cross-sectional approaches (Cole and Maxwell, 2003). By controlling for baseline exercise adherence at T1, we examine whether social support predicts *change* in adherence above and beyond stable individual differences. Additionally, the study focuses on college students in South Korea, contributing to an underrepresented literature on exercise adherence mechanisms in East Asian populations. Figure 1 presents the hypothesized model.

## 3 Method

### 3.1 Participants and procedure

Participants were recruited from three universities in South Korea using stratified random sampling to ensure representation across academic years and disciplines. Inclusion criteria were: (a) enrolled as a full-time undergraduate student, (b) aged 18–25 years, (c) able to read and write in Korean, and (d) no medical contraindications to physical activity (self-reported). Students who were enrolled in competitive varsity athletics programs were excluded to focus on recreational exercise behavior among general college students. The sample size was determined using G*Power software (Faul et al., 2009), with parameters of effect size *f*^2^ = 0.15, α = 0.05, power = 0.95, and 6 predictors, yielding a minimum required sample of 146 participants. Anticipating approximately 15% attrition across the three waves, we targeted an initial sample of 1,000 participants.

Data collection occurred across three waves during the spring semester of 2024. At T1 (baseline), participants completed measures of social support, exercise adherence, and demographic variables. At T2 (2 weeks after baseline), participants completed measures of exercise self-efficacy and athletic identity. At T3 (4 to 6 weeks after baseline), participants completed measures of intrinsic motivation and exercise adherence. This design allowed us to control for baseline levels of exercise adherence and test time-lagged mediation effects.

The initial sample at T1 comprised 950 college students. At T2, 880 participants were retained (92.6% retention rate), and at T3, 817 participants completed all measures (86.0% overall retention rate). Among the final sample (*N* = 817), 385 were male (47.1%) and 432 were female (52.9%). Participants' academic years were distributed as follows: freshmen (26.6%), sophomores (34.1%), juniors (24.0%), and seniors (15.3%). Academic majors included humanities (20.4%), natural sciences (21.8%), engineering (33.0%), medical sciences (15.4%), and arts/sports (9.3%). The mean age was 20.3 years (*SD* = 1.4).

Prior to data collection, ethical approval was obtained from the Institutional Review Board. All participants provided written informed consent after receiving detailed explanations of the study's purpose, procedures, and their rights as research participants. Anonymity and confidentiality were ensured throughout the study.

#### 3.1.1 Measurement translation and cultural adaptation

All measures originally developed in English (Self-Efficacy for Exercise Scale) were translated into Korean following standard back-translation procedures (Brislin, 1970). The translation process involved: (1) forward translation by two bilingual researchers independently; (2) reconciliation of discrepancies through discussion; (3) back-translation by an independent bilingual translator unfamiliar with the original instruments; and (4) comparison of the back-translated and original versions by the research team to identify and resolve semantic discrepancies. The translated instruments were then pilot-tested with a sample of 50 Korean college students (not included in the main study) to assess comprehension and cultural appropriateness. Minor wording adjustments were made based on participant feedback. The Social Support Scale (Ye, 2008), Exercise Adherence Scale (Wang et al., 2016), and Athletic Identity items (Dong, 2017) were originally developed in Chinese and were adapted for Korean use through a similar forward-backward translation process, given the cultural proximity but linguistic differences between Chinese and Korean populations.

### 3.2 Measures

#### 3.2.1 Social support (T1)

Social support was measured using the 14-item College Student Social Support Scale developed by (Ye 2008). Items assess perceived support from family, friends, and peers (e.g., “Most of my classmates care about me”; “I often receive emotional encouragement and support from friends”). Responses were provided on a 5-point Likert scale (1 = *strongly disagree* to 5 = *strongly agree*). Higher scores indicate greater perceived social support. Cronbach's α in the present study was 0.802.

#### 3.2.2 Exercise adherence (T1 and T3)

Exercise adherence was assessed using the 14-item Exercise Adherence Scale (EAS) developed by (Wang et al. 2016). The scale comprises three dimensions: behavioral habits (e.g., “I participate in physical exercise at least three times per week”), effort (e.g., “I am determined to persist in physical exercise”), and emotional experience (e.g., “I enjoy the feelings that physical exercise brings”). Responses were provided on a 5-point Likert scale (1 = *strongly disagree* to 5 = *strongly agree*). Cronbach's α was 0.844 at T1 and 0.862 at T3.

#### 3.2.3 Exercise self-efficacy (T2)

Exercise self-efficacy was measured at T2 using the 9-item Self-Efficacy for Exercise Scale (SEE-C) adapted by (Lee et al. 2009). Items assess confidence in maintaining exercise under challenging circumstances (e.g., “When the weather bothers you”; “When you feel tired”). Responses were provided on an 11-point scale (0 = *not confident at all* to 10 = *very confident*). Higher scores indicate greater exercise self-efficacy. Cronbach's α was 0.825.

#### 3.2.4 Athletic identity (T2)

Exercise-related identity was measured at T2 using 4 items adapted from the Sport Commitment Scale (Dong, 2017). These items assess personal meaning, pride, and psychological investment in the exerciser role (e.g., “Physical exercise is a meaningful activity for me”; “I am proud to be a sports participant”). Responses were provided on a 5-point Likert scale (1 = *strongly disagree* to 5 = *strongly agree*). Cronbach's α was 0.736.

#### 3.2.5 Intrinsic motivation (T3)

Intrinsic motivation was measured using 12 items from the intrinsic motivation sub-scale of the Exercise Motivation Scale (EMS), which was originally developed by (Li 1999) as a 31-item instrument measuring multiple dimensions of exercise motivation. For the present study, only the 12-item intrinsic motivation sub-scale was administered, as this dimension was most relevant to the theoretical model. Items assess engagement in exercise for inherent satisfaction (e.g., “Because this sport makes me feel excited and pleasant”; “Because I feel good about myself when doing this sport”). Responses were provided on a 7-point Likert scale (1 = *not at all true* to 7 = *very true*). Cronbach's α was 0.818.

### 3.3 Data analysis

Data analyses were conducted using IBM SPSS 26.0 and Mplus 8.3. The analytical procedure followed several steps. First, we examined attrition patterns by comparing T1 variables between participants who completed all waves vs. those who dropped out, using independent samples *t*-tests and chi-square tests. Second, descriptive statistics and bivariate correlations were computed for all study variables. Third, the measurement model was validated using confirmatory factor analysis (CFA), examining factor loadings, composite reliability (CR), and average variance extracted (AVE) for convergent validity, and the Fornell-Larcker criterion for discriminant validity (Fornell and Larcker, 1981). Fourth, common method bias was assessed using Harman's single-factor test and the unmeasured latent method factor approach.

The hypothesized relationships were tested using a time-lagged mediation model within the structural equation modeling (SEM) framework with maximum likelihood estimation. This approach included: (1) an autoregressive path to control for stability in exercise adherence (T1 → T3); (2) temporal paths representing the hypothesized mediation pathways; and (3) within-time correlations among variables measured at the same time point (Cole and Maxwell, 2003). Model fit was evaluated using standard indices: chi-square (χ^2^), Comparative Fit Index (CFI > 0.90), Tucker-Lewis Index (TLI > 0.90), Root Mean Square Error of Approximation (RMSEA < 0.08), and Standardized Root Mean Square Residual (SRMR < 0.08) (Hu and Bentler, 1999).

Statistical significance was evaluated at *p* < 0.05 for all analyses. Mediation effects were tested using the bias-corrected bootstrap method with 5,000 resamples (Hayes, 2009). Indirect effects were considered statistically significant if the 95% confidence interval did not include zero.

Regarding missing data, the primary analyses were conducted using the final sample of 817 participants who completed all three waves. As reported in the attrition analysis below, no significant differences were found between completers and non-completers on T1 variables, supporting the assumption of missing at random (MAR). Maximum likelihood estimation in Mplus 8.3 was used, which provides unbiased estimates under MAR conditions. As a sensitivity check, we also conducted analyses using full information maximum likelihood (FIML) estimation with the full baseline sample (*N* = 950); results were substantively identical to those reported below.

## 4 Results

### 4.1 Preliminary analyses

#### 4.1.1 Attrition analysis

To assess potential attrition bias, we compared participants who completed all three waves (*n* = 817) with those who dropped out (*n* = 133) on T1 variables. Independent samples *t*-tests revealed no significant differences in social support (*t* = –0.28, *p* = 0.780, Cohen's *d* = –0.03) or baseline exercise adherence (*t* = 0.85, *p* = 0.396, Cohen's *d* = 0.08). Chi-square tests indicated no significant differences in gender (χ^2^ = 1.18, *p* = 0.277) or academic year (χ^2^ = 2.54, *p* = 0.468) between completers and non-completers. The small effect sizes suggest that attrition was not systematically related to key study variables, supporting the assumption of missing at random (MAR).

#### 4.1.2 Measurement model validation

Prior to testing the structural model, we examined the measurement properties of all scales using confirmatory factor analysis (CFA). The measurement model including all latent variables demonstrated acceptable fit: χ^2^(df) = 1,087.53 (624), CFI = 0.956, TLI = 0.950, RMSEA = 0.030, SRMR = 0.038. All standardized factor loadings were significant and ranged from 0.60 to 0.88, exceeding the recommended threshold of 0.50 (Hair et al., 1992).

Table 1 presents the reliability and validity indices for all study measures. Cronbach's α coefficients ranged from 0.736 to 0.862, all exceeding the 0.70 threshold for acceptable internal consistency (Nunnally, 1978). Composite reliability (CR) values ranged from 0.74 to 0.86 (all > 0.70), indicating adequate construct reliability. Average variance extracted (AVE) values ranged from 0.50 to 0.52 (all ≥ 0.50), indicating adequate convergent validity (Fornell and Larcker, 1981).

**Table 1:** Reliability and convergent validity of study measures.

Scale	Time	Items	α	CR	AVE	Factor Loadings
Social support scale	T1	14	0.802	0.81	0.50	0.60–0.82
Exercise adherence scale	T1	14	0.844	0.85	0.51	0.61–0.83
Self-efficacy for exercise scale	T2	9	0.825	0.83	0.51	0.62–0.80
Athletic identity scale	T2	4	0.736	0.74	0.50	0.63–0.78
Intrinsic motivation scale	T3	12	0.818	0.82	0.51	0.60–0.79
Exercise adherence scale	T3	14	0.862	0.86	0.52	0.62–0.85

α, Cronbach's alpha; CR, composite reliability; AVE, average variance extracted. All factor loadings were significant at *p* < 0.001.

Discriminant validity was assessed using the Fornell-Larcker criterion. As shown in Table 2, the square root of AVE for each construct (diagonal values) exceeded its correlations with all other constructs (off-diagonal values), supporting discriminant validity. Additionally, all heterotrait-monotrait (HTMT) ratios were below 0.85, further confirming discriminant validity.

**Table 2:** Discriminant validity: square root of AVE and inter-construct correlations.

Variable	1	2	3	4	5	6
1. T1 Social support	**0.71**					
2. T1 Exercise adherence	0.13	**0.71**				
3. T2 Self-efficacy	0.22	0.23	**0.71**			
4. T2 Athletic identity	0.17	0.17	0.45	**0.71**		
5. T3 Intrinsic motivation	0.15	0.23	0.45	0.32	**0.71**	
6. T3 Exercise adherence	0.12	0.49	0.34	0.22	0.36	**0.72**

Bold diagonal values are the square root of AVE; off-diagonal values are inter-construct correlations. Discriminant validity is supported when diagonal values exceed off-diagonal values in corresponding rows and columns.

#### 4.1.3 Common method bias

Although the longitudinal design with temporally separated measurements provides procedural control for common method bias, we conducted additional statistical tests. First, Harman's single-factor test on T1 data revealed that the first unrotated factor explained 21.9% of the variance, well below the 40% threshold (Podsakoff et al., 2003). Second, we tested an unmeasured latent method factor model, wherein adding a common method factor did not substantially improve model fit (ΔCFI = 0.006), and the factor loadings on the method factor were nonsignificant or negligible. These results suggest that common method bias is not a serious concern in this study.

### 4.2 Descriptive statistics and correlations

Descriptive statistics for all study variables are presented in Table 3. All variables demonstrated acceptable skewness values (range: –0.50 to 0.20), well within acceptable limits for maximum likelihood estimation (Kline, 2015). The means for all psychological variables were above the scale midpoints, suggesting generally positive levels of social support, self-efficacy, athletic identity, intrinsic motivation, and exercise adherence among participants.

**Table 3:** Descriptive statistics for study variables.

Variable	*N*	*M*	*SD*	Min	Max	Skewness
T1 Social support	950	3.15	0.53	1.43	4.38	–0.42
T1 Exercise adherence	950	2.92	0.57	1.57	4.43	0.22
T2 Self-efficacy	880	5.50	1.51	0.56	9.33	–0.32
T2 Athletic identity	880	3.27	0.74	1.00	5.00	–0.38
T3 Intrinsic motivation	817	4.40	0.83	1.82	6.42	–0.50
T3 Exercise adherence	817	2.97	0.59	1.36	4.64	0.06

Self-efficacy was measured on a 0–10 scale; intrinsic motivation on a 1–7 scale; all other variables on a 1–5 scale.

Bivariate correlations among study variables are presented in Table 4. Consistent with theoretical expectations, T1 social support was significantly positively correlated with T2 self-efficacy (*r* = 0.22, *p* < 0.001), T2 athletic identity (*r* = 0.17, *p* < 0.001), T3 intrinsic motivation (*r* = 0.15, *p* < 0.001), and T3 exercise adherence (*r* = 0.12, *p* < 0.001).

**Table 4:** Bivariate correlations among study variables (*N* = 817).

Variable	1	2	3	4	5	6
1. T1 Social support	—					
2. T1 Exercise adherence	0.13^***^	—				
3. T2 Self-efficacy	0.22^***^	0.23^***^	—			
4. T2 Athletic identity	0.17^***^	0.17^***^	0.45^***^	—		
5. T3 Intrinsic motivation	0.15^***^	0.23^***^	0.45^***^	0.32^***^	—	
6. T3 Exercise adherence	0.12^***^	0.49^***^	0.34^***^	0.22^***^	0.36^***^	—

^*^p < 0.05. ^**^p < 0.01. ^***^p < 0.001.

T1 exercise adherence demonstrated a strong correlation with T3 exercise adherence (*r* = 0.49, *p* < 0.001), underscoring the importance of controlling for baseline levels when examining temporal relationships. The two T2 mediators were significantly correlated (*r* = 0.45, *p* < 0.001). T2 self-efficacy was significantly correlated with T3 intrinsic motivation (*r* = 0.45, *p* < 0.001) and T3 exercise adherence (*r* = 0.34, *p* < 0.001). T2 athletic identity was also significantly correlated with T3 intrinsic motivation (*r* = 0.32, *p* < 0.001) and T3 exercise adherence (*r* = 0.22, *p* < 0.001).

### 4.3 Time-lagged mediation analysis

#### 4.3.1 Model specification and fit

We tested a time-lagged mediation model that included: (1) an autoregressive path for exercise adherence (T1 → T3); (2) temporal paths representing the hypothesized mediation pathways; and (3) within-time correlations among variables measured at the same time point. This approach allows for rigorous testing of temporal precedence while controlling for stability in exercise adherence over time (Cole and Maxwell, 2003).

The time-lagged structural model demonstrated good fit to the data: χ^2^(df) = 298.36(98), *p* < 0.001, CFI = 0.965, TLI = 0.956, RMSEA = 0.050 (90% CI [0.043, 0.057]), SRMR = 0.043. All fit indices met or exceeded recommended thresholds (Hu and Bentler, 1999), indicating that the hypothesized model adequately represented the observed data.

#### 4.3.2 Autoregressive effects

Exercise adherence showed strong stability from T1 to T3 (β = 0.41, *SE* = 0.03, *p* < 0.001). This substantial autoregressive effect underscores the importance of controlling for baseline levels when examining longitudinal relationships, as a considerable proportion of the variance in T3 exercise adherence was explained by T1 exercise adherence.

#### 4.3.3 Path coefficients

**Figure 2:**
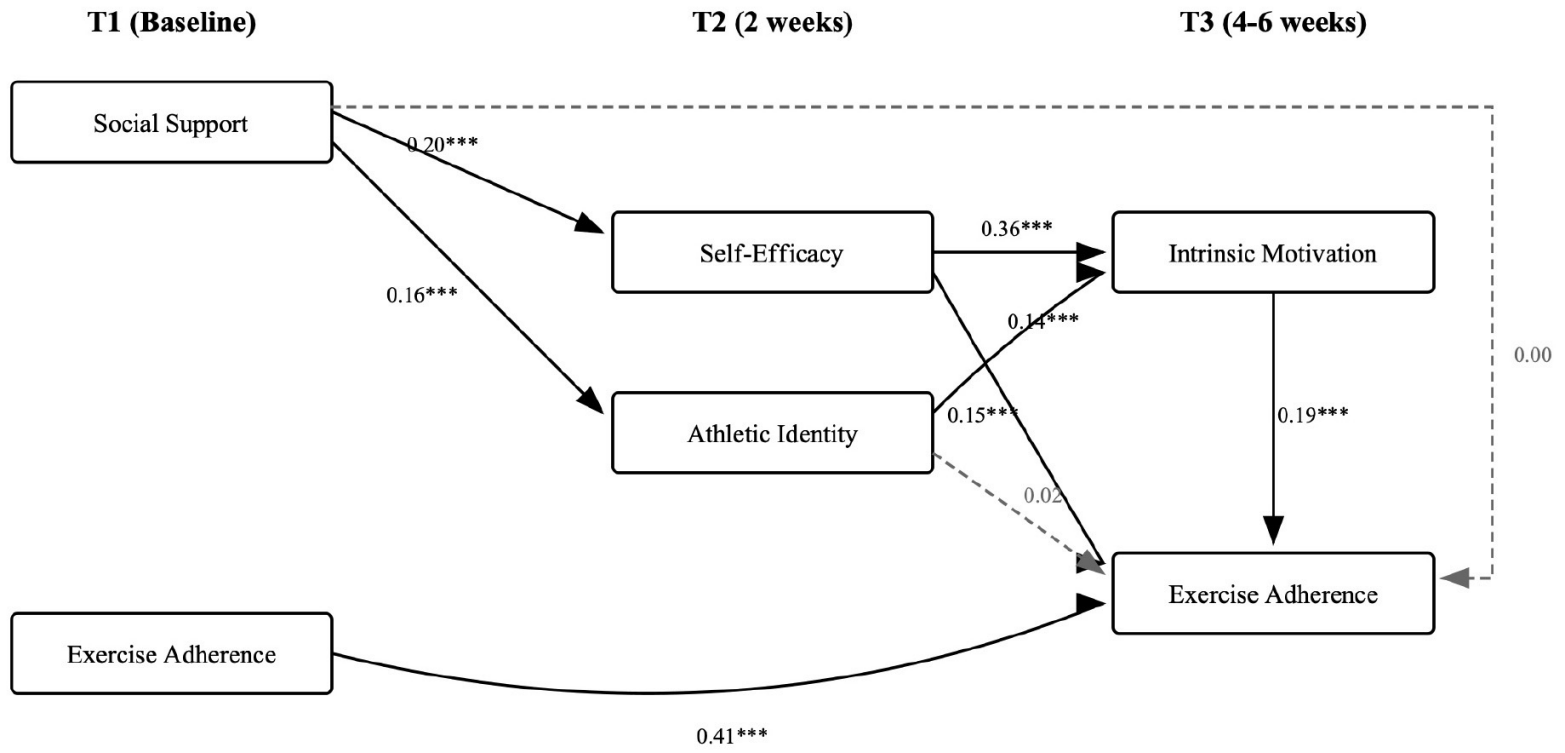
Standardized path coefficients for the time-lagged mediation model. Solid lines represent significant paths; dashed lines represent non-significant paths. ^**^p < 0.01. ^***^p < 0.001.

Figure 2 presents the standardized path coefficients for the structural model. After controlling for baseline exercise adherence, T1 social support positively predicted T2 self-efficacy (β = 0.20, *SE* = 0.03, *p* < 0.001) and T2 athletic identity (β = 0.16, *SE* = 0.03, *p* < 0.001). T2 self-efficacy positively predicted T3 intrinsic motivation (β = 0.36, *SE* = 0.04, *p* < 0.001) and T3 exercise adherence (β = 0.15, *SE* = 0.04, *p* < 0.001). T2 athletic identity significantly predicted T3 intrinsic motivation (β = 0.14, *SE* = 0.04, *p* < 0.001) but did not significantly predict T3 exercise adherence (β = 0.02, *SE* = 0.03, *p* = 0.57). T3 intrinsic motivation positively predicted T3 exercise adherence (β = 0.19, *SE* = 0.03, *p* < 0.001). The direct effect of T1 social support on T3 exercise adherence was negligible (β = 0.00, *SE* = 0.03, *p* = 0.92), consistent with an indirect-only mediation pattern (Zhao et al., 2010).

#### 4.3.4 Direct and indirect effects

The results of the mediation analysis using bias-corrected bootstrap method (5,000 resamples) are summarized in Table 5. The total effect of T1 social support on T3 exercise adherence was small and did not reach conventional significance (β = 0.058, *SE* = 0.032, 95% CI [–0.005, 0.119], *p* = 0.061). The direct effect was negligible (β = 0.003, *SE* = 0.030, 95% CI [–0.056, 0.060], *p* = 0.92). However, the total indirect effect was significant (β = 0.050, *SE* = 0.011, 95% CI [0.030, 0.073]). This pattern is consistent with “indirect-only mediation” (Zhao et al., 2010), wherein indirect effects are significant despite a non-significant total effect. The modest total effect likely reflects the strong autoregressive stability of exercise adherence (β = 0.41), which absorbs substantial variance, along with shared variance among mediators (Hayes, 2009; Rucker et al., 2011).

**Table 5:** Bootstrap results for direct and indirect effects.

Effect	Estimate	SE	95% CI
**Total effect**	0.058	0.032	[–0.005, 0.119]
**Direct effect**	0.003	0.030	[–0.056, 0.060]
**Total indirect effect**	0.050	0.011	[0.030, 0.073]
Specific indirect effects			
SS → SE → EA	0.029	0.009	[0.013, 0.049]
SS → AI → EA	0.003	0.005	[–0.007, 0.014]
SS → SE → IM → EA	0.013	0.004	[0.007, 0.021]
SS → AI → IM → EA	0.004	0.002	[0.002, 0.008]

SS, Social support; SE, Self-efficacy; AI, Athletic identity; IM, Intrinsic motivation; EA, Exercise adherence. Bootstrap resamples = 5,000. Confidence intervals excluding zero indicate significant effects.

Four indirect pathways were tested. Three pathways were statistically significant:

**Pathway 1 (H2): Social support** **→** **Self-efficacy** **→** **Exercise adherence**. The indirect effect through self-efficacy was significant (effect = 0.029, *SE* = 0.009, 95% CI [0.013, 0.049]), representing the strongest indirect pathway. This finding supports Hypothesis 2, indicating that self-efficacy is a primary mechanism through which social support influences exercise adherence.

**Pathway 2 (H3): Social support** **→** **Athletic identity** **→** **Exercise adherence**. The indirect effect through athletic identity was not significant (effect = 0.003, *SE* = 0.005, 95% CI [–0.007, 0.014]). This finding does not support Hypothesis 3, suggesting that athletic identity does not serve as an independent mediator linking social support to exercise adherence in this sample.

**Pathway 3 (H4a): Social support** **→** **Self-efficacy** **→** **Intrinsic motivation** **→** **Exercise adherence**. The serial mediation effect through self-efficacy and intrinsic motivation was significant (effect = 0.013, *SE* = 0.004, 95% CI [0.007, 0.021]). This finding supports Hypothesis 4a, demonstrating a sequential pathway from environmental support through cognitive resources to motivational processes to behavioral outcomes.

**Pathway 4 (H4b): Social support** **→** **Athletic identity** **→** **Intrinsic motivation** **→** **Exercise adherence**. The serial mediation effect through athletic identity and intrinsic motivation was significant (effect = 0.004, *SE* = 0.002, 95% CI [0.002, 0.008]). This finding supports Hypothesis 4b, indicating that athletic identity contributes to exercise adherence through its effect on intrinsic motivation, rather than directly.

The total indirect effect was 0.050 (*SE* = 0.011, 95% CI [0.030, 0.073]). Given the marginal total effect, proportions mediated should be interpreted cautiously; however, the indirect effects were clearly the dominant pathway linking social support to exercise adherence. The significant indirect effects operated primarily through self-efficacy pathways, with the self-efficacy-related pathways (0.029 + 0.013 = 0.042) representing the substantial majority of the total indirect effect. While athletic identity did not directly predict exercise adherence, it contributed through intrinsic motivation, supporting a more nuanced role for identity in the mediation process.

#### 4.3.5 Summary of hypothesis testing

In summary, Hypotheses 2, 4a, and 4b were supported, while Hypotheses 1 and 3 were not fully supported. The total effect of social support on exercise adherence was marginal (H1: β = 0.058, *p* = 0.061), but the total indirect effect was significant, indicating that social support operates through the proposed mediators rather than directly. Self-efficacy emerged as the primary mediator (H2 supported), whereas athletic identity did not serve as an independent mediator (H3 not supported). Both serial mediation pathways through intrinsic motivation were significant (H4a and H4b supported). Table 5 provides the complete bootstrap results.

## 5 Discussion

This three-wave longitudinal study found that social support influences exercise adherence among Korean college students primarily through self-efficacy-based pathways, with athletic identity contributing indirectly through intrinsic motivation. Significant indirect effects emerged despite a modest total effect, consistent with an indirect-only mediation pattern (Zhao et al., 2010). Below, we discuss these findings in relation to prior research and their theoretical and practical implications.

### 5.1 Social support and exercise adherence

The total effect of social support on exercise adherence was small (β = 0.058, *p* = 0.061) and did not reach conventional significance thresholds. However, this finding should be interpreted in light of contemporary mediation theory, which recognizes that significant indirect effects can emerge even when the total effect is modest or non-significant (Hayes, 2009; Zhao et al., 2010; Rucker et al., 2011). The total indirect effect was significant (β = 0.050, 95% CI [0.030, 0.073]), while the direct effect was negligible (β = 0.003, *p* = 0.92). This pattern is consistent with “indirect-only mediation,” suggesting that social support is linked to exercise adherence primarily through the proposed mediating mechanisms rather than through direct effects.

The positive association between social support and exercise adherence aligns with Social Cognitive Theory (Bandura, 1986) and is consistent with findings from both observational and experimental research. Longitudinal studies have documented that social support predicts subsequent physical activity behavior (Tian and Shi, 2022; Liu W. et al., 2024), and RCTs have shown that interventions incorporating social support components produce meaningful increases in physical activity (Dishman et al., 2005; Lubans et al., 2008). The indirect-only mediation pattern observed in the present study—where social support operates through cognitive and motivational mechanisms rather than directly—is consistent with SCT's core proposition and extends prior findings by demonstrating this pattern with temporally separated measurements.

The finding that social support influences exercise adherence through mediating pathways, rather than merely correlating with stable individual differences, has important implications. This finding is consistent with the possibility that interventions targeting both social support enhancement and psychological mediators may be more effective than targeting social support alone. This conclusion is strengthened by the inclusion of baseline exercise adherence as a control variable, which absorbs variance attributable to stable individual characteristics and prior exercise history.

### 5.2 Mediating role of self-efficacy

Self-efficacy emerged as the primary mediator, with self-efficacy-related pathways accounting for the substantial majority (84%) of the total indirect effect. This finding is consistent with prior cross-sectional research (Ke et al., 2025; Sánchez-García et al., 2024), meta-analytic evidence on self-efficacy and physical activity (Ashford et al., 2010), and experimental studies demonstrating that self-efficacy interventions increase physical activity behavior (McAuley et al., 2011). The present study extends these converging findings through a temporally structured design, demonstrating that social support at T1 predicted self-efficacy at T2, which in turn predicted exercise adherence at T3.

These findings are consistent with SCT's specification of self-efficacy sources—mastery experiences, vicarious learning, verbal persuasion, and physiological states (Bandura, 1986)—each of which can be enhanced through social support. Experimental evidence corroborates these mechanisms: RCTs have shown that structured programs providing mastery experiences through graded exercise, peer modeling, and instructor encouragement effectively increase self-efficacy and subsequent physical activity (McAuley et al., 2011; Ashford et al., 2010). In the present study, social support likely operated through these same channels—providing verbal encouragement, modeling exercise behavior, facilitating successful exercise experiences, and buffering stress (Caetano et al., 2020; Schunk and DiBenedetto, 2020).

Enhanced self-efficacy then promotes exercise adherence through multiple downstream mechanisms. Individuals with high exercise self-efficacy set more challenging goals for physical activity, exert greater effort when facing obstacles such as fatigue or time constraints, develop more effective self-regulatory strategies for maintaining exercise routines, and recover more quickly from lapses or setbacks (Lee et al., 2009). The significant path coefficient from self-efficacy to exercise adherence (β = 0.15) underscores the role of efficacy beliefs in sustaining health behaviors, consistent with SCT's proposition that self-efficacy is the foundation of human agency (Bandura, 2004).

### 5.3 Non-significant role of athletic identity

Athletic identity did not serve as an independent mediator linking social support to exercise adherence, though it contributed indirectly through intrinsic motivation. This finding is noteworthy given that prior research with competitive athletes has documented stronger direct identity-behavior links (Strachan et al., 2010; Castillo et al., 2020). The weaker direct effect in our general college student sample likely reflects that athletic identity is less central to these individuals' self-concepts compared to dedicated athletes (Dong, 2017). In general populations, identity may function primarily as a motivational catalyst—making exercise feel personally meaningful and self-congruent—rather than directly driving behavioral consistency. Additionally, the substantial shared variance between self-efficacy and athletic identity (*r* = 0.45) suggests that when both are included in the model, self-efficacy captures the variance more directly related to behavioral outcomes. These findings align with research indicating that identity-behavior links are moderated by the centrality of the identity to the individual's self-concept (Strachan et al., 2010).

### 5.4 Serial mediation through self-efficacy and intrinsic motivation

Both serial mediation pathways through intrinsic motivation were significant, demonstrating that self-efficacy and athletic identity contribute to exercise adherence partly through their effects on autonomous motivation. These findings align with SCT's proposition that self-efficacy constitutes the foundation for motivation and action (Bandura, 2004), and with self-determination theory's emphasis on intrinsic motivation as the most sustainable form of behavioral regulation (Castillo et al., 2020). Importantly, the sequential pattern—from environmental support through psychological resources to motivational processes to behavior—is consistent with experimental evidence showing that efficacy-enhancing interventions produce downstream effects on motivation and subsequent physical activity (McAuley et al., 2011). The finding that identity influences adherence through motivation rather than directly is theoretically consistent with the notion that identity provides psychological meaning to activities, which manifests behaviorally through enhanced motivation (Strachan et al., 2010). This temporal ordering suggests that interventions targeting both self-efficacy and athletic identity may produce synergistic downstream benefits for motivation and adherence.

### 5.5 Theoretical implications

The present findings advance understanding of SCT-based mediation processes in several ways. First, the time-lagged design provides evidence consistent with the temporal ordering proposed by SCT (Cole and Maxwell, 2003), though with an important caveat: because intrinsic motivation and exercise adherence were assessed concurrently at T3, the final link in the mediation chain (IM → EA) represents a model-implied sequence rather than established temporal precedence. Causal conclusions therefore remain tentative, and experimental manipulation would be needed to establish definitive causation.

Second, the “indirect-only mediation” pattern (Zhao et al., 2010) supports SCT's proposition that environmental factors influence behavior through cognitive and motivational mediators. It is important to note, however, that while the indirect effects were statistically significant, their absolute magnitudes were small (total indirect effect = 0.050). This is consistent with the expectation that any single environmental factor will have modest effects on a complex, multiply-determined behavior like exercise adherence, particularly after controlling for baseline behavior. The practical significance of these effects should be interpreted in the context of population-level interventions, where even small effects can have meaningful public health impact when applied across large populations (Rucker et al., 2011).

Third, the moderate stability in exercise adherence (β = 0.41) indicates that while prior behavior is the strongest predictor, there remains substantial variance that can be influenced by psychological factors during the college years.

### 5.6 Practical implications

The findings yield several concrete recommendations for promoting exercise adherence among college students. First, because self-efficacy emerged as the dominant mediator, campus exercise programs should be specifically designed to build efficacy beliefs. Concrete strategies include: (a) implementing “exercise ladder” programs that start with low-intensity activities (e.g., 10-min walking groups) and progressively increase in duration and intensity, ensuring early mastery experiences; (b) pairing inexperienced students with trained peer exercise mentors who model successful routines and provide encouragement; (c) using app-based or in-person self-monitoring tools that provide real-time feedback on progress to reinforce competence perceptions; and (d) offering diverse exercise modalities (yoga, dance, martial arts, and team sports) so students can find activities matching their interests and ability levels (Lee et al., 2009; McAuley et al., 2011).

Second, social support interventions should be structured rather than generic. Specific implementation strategies include: establishing exercise buddy matching programs based on schedule compatibility and fitness level; creating campus-wide physical activity challenges with team-based accountability structures; training resident advisors to conduct weekly floor-based exercise sessions; and developing family engagement protocols (e.g., shared fitness tracking between students and family members) (Mema et al., 2022; Tian and Shi, 2022; Dishman et al., 2005).

Third, the serial mediation pathway through intrinsic motivation suggests that fostering enjoyment is critical for long-term adherence. Practitioners should offer “exercise sampling” periods at the start of each semester where students try multiple activities before committing, use autonomy-supportive instructional approaches that allow students to choose their exercise format and intensity, and incorporate gamification elements that enhance the inherent enjoyment of physical activity (Li et al., 2024b; Chen et al., 2013).

Fourth, while athletic identity did not directly predict adherence, identity-based approaches may benefit specific subgroups. Exercise professionals could implement identity-strengthening strategies—such as exercise-related social media communities, campus exercise clubs with visible branding, and recognition programs for exercise milestones—particularly targeting students with some prior athletic experience who may be most receptive to identity-based interventions.

### 5.7 Limitations and future directions

Several limitations warrant consideration. First, although the longitudinal design improves upon cross-sectional approaches, causal conclusions remain tentative without experimental manipulation; future randomized trials testing interventions targeting self-efficacy could strengthen causal inferences. Second, the sample was limited to Korean college students, and replication across diverse cultural and age groups is needed. Third, intrinsic motivation and exercise adherence were assessed concurrently at T3, limiting inferences about this final pathway; four-wave designs could establish clearer temporal ordering. Fourth, all measures were self-reported, and future research incorporating objective physical activity measures (e.g., accelerometry) would enhance validity. Finally, exercise-related identity was assessed with a brief measure adapted from a commitment scale, which may not fully capture identity constructs; more comprehensive measures warrant investigation.

## 6 Conclusions

This three-wave longitudinal study advances understanding of how social support influences exercise adherence among college students by identifying the psychological mechanisms underlying this relationship. The findings reveal an indirect-only mediation pattern: while the total effect of social support on exercise adherence was modest, significant indirect effects emerged through self-efficacy and intrinsic motivation pathways. Self-efficacy served as the dominant mediator, both directly influencing exercise adherence and indirectly through intrinsic motivation. Exercise-related identity contributed through its association with intrinsic motivation rather than directly predicting behavior, suggesting that identity-based processes may require motivational translation to influence behavioral outcomes.

These findings carry theoretical and practical significance. Theoretically, the results support Social Cognitive Theory's core proposition that environmental factors influence behavior through cognitive mediators, with self-efficacy playing a central role. Practically, the findings suggest that campus health promotion programs should prioritize building exercise self-efficacy through mastery experiences, social modeling, and supportive feedback, while simultaneously fostering intrinsic motivation through autonomy support and enjoyable exercise experiences. Interventions that strengthen social support networks while developing psychological resources may produce synergistic effects on sustained physical activity among college students.

## Data Availability

The raw data supporting the conclusions of this article will be made available by the authors, without undue reservation.
